# A method for the construction of equalized directional cDNA libraries from hydrolyzed total RNA

**DOI:** 10.1186/1471-2164-8-363

**Published:** 2007-10-09

**Authors:** Claytus Davis, Zeev Barvish, Inna Gitelman

**Affiliations:** 1 Department of Virology and Developmental Genetics, Faculty of Health Science, Ben Gurion University of the Negev, Beer Sheva, Israel

## Abstract

**Background:**

The transcribed sequences of a cell, the transcriptome, represent the trans-acting fraction of the genetic information, yet eukaryotic cDNA libraries are typically made from only the poly-adenylated fraction. The non-coding or translated but non-polyadenylated RNAs are therefore not represented. The goal of this study was to develop a method that would more completely represent the transcriptome in a useful format, avoiding over-representation of some of the abundant, but low-complexity non-translated transcripts.

**Results:**

We developed a combination of self-subtraction and directional cloning procedures for this purpose. Libraries were prepared from partially degraded (hydrolyzed) total RNA from three different species. A restriction endonuclease site was added to the 3' end during first-strand synthesis using a directional random-priming technique. The abundant non-polyadenylated rRNA and tRNA sequences were largely removed by using self-subtraction to equalize the representation of the various RNA species. Sequencing random clones from the libraries showed that 87% of clones were in the forward orientation with respect to known or predicted transcripts. 70% matched identified or predicted translated RNAs in the sequence databases. Abundant mRNAs were less frequent in the self-subtracted libraries compared to a non-subtracted mRNA library. 3% of the sequences were from known or hypothesized ncRNA loci, including five matches to miRNA loci.

**Conclusion:**

We describe a simple method for making high-quality, directional, random-primed, cDNA libraries from small amounts of degraded total RNA. This technique is advantageous in situations where a cDNA library with complete but equalized representation of transcribed sequences, whether polyadenylated or not, is desired.

## Background

Almost the entire trans-acting fraction of genetic information is represented by the transcriptome, the population of transcribed sequences in a cell. In terms of complexity, much of the functional transcriptome of eukaryotic cells has traditionally been considered poly-adenylated and translated. In terms of quantity, this poly-adenylated fraction constitutes only 3–6% of the total RNA population. For these reasons, experimental representation of eukaryotic transcriptomes was usually done by constructing cDNA libraries from the poly A^+ ^fraction of the RNA population. All such libraries do not, by design, represent the entire trans-acting genetic information. They lack representation of non-coding but functional RNAs (ncRNA), e.g. [1], including the abundant but low complexity tRNAs and rRNAs and the increasingly studied populations of various snRNAs, scRNAs, snoRNAs, telomeric RNAs, vRNAs, and microRNAs [2-7]. They lack representation of the mRNAs of organelles – mitochondria and chloroplasts – for which polyadenylation may be a signal for degradation [8,9]. They lack representation of mRNAs that are not poly-adenylated or lose their polyA tails, but are nevertheless translated [10-13]. The recent call for a more systematic examination of the entire transcriptome – RNomics [14] led to much greater interest in ncRNAs and a variety of wet and computational approaches to their identification [reviewed in [15,16]].

Our purpose here was to develop a library construction method that would result in a more complete representation, in useable form, of the transcriptome. We reasoned that self-subtraction [17,18], which equalizes the representation of different sequences through reassociation kinetics, should work as well as poly A^+ ^selection for reducing the frequency of the abundant but low-complexity rRNAs and tRNAs, without eliminating them, or any other polyA^- ^RNAs, from cDNA libraries. We describe here the method and show that informative, random-primed, directional, and more completely representative cDNA libraries can be made from partially degraded total RNA.

## Results and discussion

### RNA preparation

Since a method for the production of high-quality, more fully representative cDNA libraries from even difficult samples was sought, three very different RNA sources were chosen: 48 hour zebrafish (*Danio rerio*) embryos, field-collected 36 hour embryonic amphioxus (*Branchiostoma floridae*), and isolated 3rd instar fruitfly (*Drosophila melanogaster*) larval brain and eye discs. Total RNA was extracted from these samples with TRIzol (Invitrogen, manufacturer's protocol). Contaminating genomic DNA was completely removed from aliquots of the RNAs by digestion with 1 U RNase-free DNase (NewEngland Biolabs)/ug RNA in the manufacturer's buffer for 30 minutes at room temperature. 1 μg of RNase free glycogen (Roche) was then added and the sample re-extracted with Trizol (Methods-1). Total RNA was partially hydrolyzed in 100 m**M **(Na)CO_3_, pH 10.0, for 20 min. at 60°C. This resulted in a population of 100–1300 nt RNA fragments (Methods-2).

### cDNA synthesis

**Figure 1:**
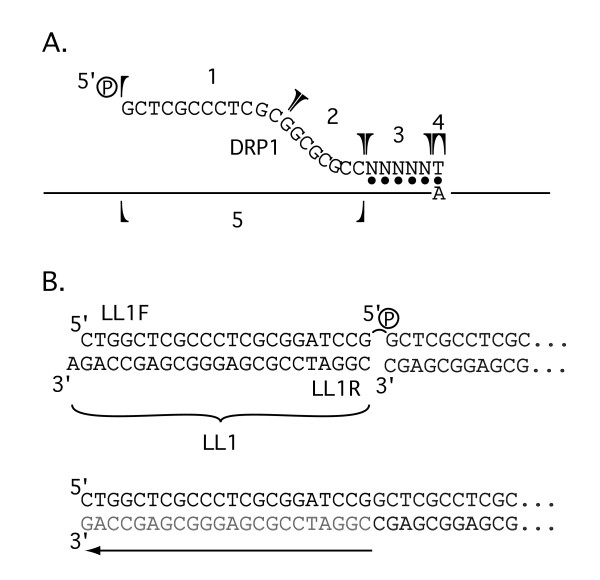
**Random directional cDNA synthesis**. **A**. The first strand cDNA primer (DRP1) contains: a 5' phosphorylated buffer sequence devoid of A bases (1), an AscI site (2), a 5 nt random sequence (3), and a 3' T residue (4). By design, priming should begin only at an A in the RNA template. **B**. The non-phosphorylated lone-linker LL1 consists of the the two complimentary oligonucleotides LL1F and LL1R. It has one blunt end and one non-adhesive staggered end. LL1 can therefore ligate only to one strand of the cDNAs and in only one orientation. The remaining nick in the second strand is removed by preincubating the cDNAs before the first PCR reaction at 72°C for one minute to strip off the non-ligated strand of the linker and regenerate the sequence by extension from the 3' end of the cDNA (lower grey).

Double-stranded (ds) cDNAs were synthesized from the partially-degraded total RNA by a standard Gubler-Hoffman replacement procedure. Primer and template were annealed by mixing 5 μg of the partially hydrolyzed, DNA-free RNA with 0.7 μg of the 5'-phosphorylated, directional, 1st-strand primer (DRP1) (Fig. 1A), heating to 70°C for 10 minutes, and quenching briefly on ice. The first strand was synthesized by incubating the annealed mix with 200 units of Superscript II reverse transcriptase (Invitrogen) for one hour at 45°C in the manufacturer's buffer containing 10 mM DTT and 0.5 mM dNTPs. The second strand was synthesized by adding 90 μl H_2_O, 32 μl 5× 2nd strand buffer [100 mM HEPES pH 6.9 (buffered with KOH), 50 mM KCl, 25 mM MgCl_2_, 50 mM (NH_4_)_2_SO_4_], 6 μl dNTPs (5 mM each), 1 μl ^®^NAD (10 mM), 2 μl *E. coli *DNA ligase (6 U/μg), 6 μl 0.1 M DTT, 4 μl *E. coli *DNA polymerase I (10 U/ul), and 1 μl *E. coli *RNase H (2 U/μl). The reaction mixture was incubated for 16 hours at 16°C. Double-stranded cDNAs were then blunt-ended by adding 10 units of T4 DNA polymerase (New England Biolabs) and continuing the incubation for 10 minutes. The blunt-ended, double-stranded cDNAs (yields were between 1.2 and 1.6 μg) were then extracted with phenol:chloroform (1:1), precipitated with ammonium acetate and ethanol and then resuspended in 10 μl of TE. Lone linker LL1 (Fig. 1B) was prepared by annealing equimolar amounts of LL1L and LL1R (Fig. 1B) for 15 minutes at room temperature in TE. 1.2 μg of LL1 was then added to the cDNA and ligated overnight at 16°C (Methods-3). Ligated cDNAs were extracted with phenol:chloroform (1:1) and precipitated with sodium acetate and ethanol and resuspended in 50 μl TE. Fragments smaller than approximately 100 bp were removed by Sepharose CL-6B (Roche) gel filtration. The 250 μl flow-through peak was collected and the cDNAs precipitated with sodium acetate, 1 μl of glycogen carrier and ethanol and resuspended in 20 μl TE. This >100 bp cDNA population was amplified in a 10 cycle PCR reaction. Each 100 μl reaction contained 1 μg of the primer LL1P, 6 U and 0.6 U of Vent exo^-^, and exo^+ ^thermostable DNA polymerases (New England Biolabs) respectively, 0.5 mM dNTPs, and 1 μl of α^32^P dCTP. An initial incubation at 72°C for one minute generated full-length 3' ends (Fig. 1B) (Methods-4). The first 7 cycles were 95°C, 55°C, and 72°C for 1 min each. In the final three cycles, the 72°C extension steps were increased to 2, 4, and 8 min. respectively. Amplified cDNAs were purified by phenol/Chloroform extraction and ethanol precipitation.

### Self-subtraction

5 μg of amplified cDNA in 10 μl of annealing buffer (0.34 **M **NaCl, 0.1 **M **Na(PO_4_) pH 6.8, 1 m**M **EDTA) containing 300 ng of LL1F was overlayed with mineral oil, denatured by boiling for 5 min and then annealed at 60°C for one hour (C_o_t ~ 3 × 10^-4 ^M·min). 100 μl of binding buffer (0.12 **M **Na(PO_4_), pH 6.8) was added to the bottom aqueous phase which was then transferred to another tube. 100 μl of hydrated hydroxylapatite (Methods-5) suspended in 1.2 ml of binding buffer at 60°C was then added and the suspension incubated at 60°C for 10 min with frequent mixing. Bound dsDNA and hydroxylapatite were removed completely by discarding the pellets after two consecutive centrifugations (Methods-6). The ^32^P counts of sample aliquots taken before and after subtraction, indicated that between 90 and 97% of the cDNA was bound to the hydroxylapatite and removed. The hydroxylapatite phosphate buffer was replaced with 10 mM Tris, 1 mM EDTA, pH8 (TE), by four consecutive exchanges in Centricon-100 filters (Amicon; Methods-7). The remaining single-stranded (ss) cDNAs were then subjected to a second round of amplification, and self-subtraction as described above, with the exception that the second reannealing time was 24 hours instead of one hour (C_o_t of ~ 7 × 10^-3 ^M·min). Approximately 80% of the cDNA amplified after the first subtraction was removed in the second self-subtraction. The phosphate buffer of the second self-subtraction reaction was exchanged for TE as described. The final double-stranded cDNA population for cloning was generated using the same PCR protocol used for the previous amplifications.

### Cloning

Regenerated ds cDNAs were then digested with *Bam *HI and *Asc *I, and size-selected by agarose gel electrophoresis to obtain 200–300 bp cDNA fractions. These were then ligated into pKE-1 or pKE-2 vectors [19] and used to transform *E. coli *DH10B (Invitrogen) by electroporation. The three cDNA libraries – amphioxus, *Drosophila *and zebrafish – contained 6 × 10^6^, 4 × 10^5^, and 3 × 10^6 ^independent clones respectively.

## Analysis

**Figure 2:**
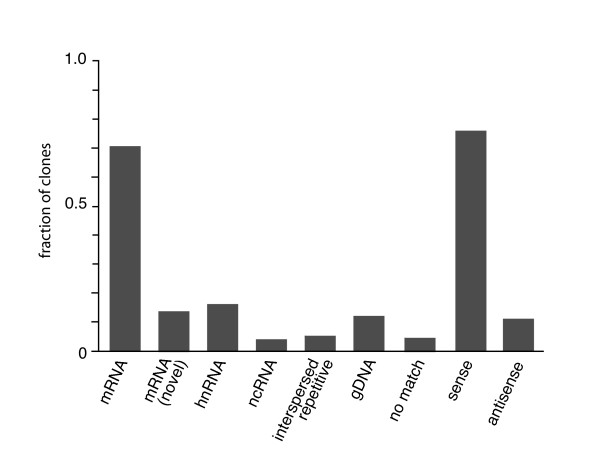
**Characterization of equalized cDNA libraries**. 257 cDNA sequences from the *Danio rerio *and *Drosophila melanogaster *self-subtracted cDNA libraries were used as GenBank and Ensembl database query sequences. Diagnostic properties of near identity (>97%) matches were collected and scored. The labeled columns represent the following library characteristics: **mRNA **– the fraction of clones matching known or hypothetical mRNA sequences. **mRNA (novel) **– the fraction of clones representing the first reported matches to hypothetical mRNA sequence or previously unknown splice variants of known mRNA transcription units. **hnRNA **– the fraction of clones matching sequence that is likely removed during mRNA processing, eg. intron. **ncRNA **– the fraction of clones matching known or hypothetical non-coding RNA transcription units. **interspersed repetitive **– the fraction of clones matching more than 50 dispersed loci in the genome with probabilities of e < 10^-9^. **gDNA **– the fraction of clones matching genomic DNA outside of any known or predicted transcription unit. **no match **– the fraction of clones that did not match any known cDNA and could not be assigned an origin in the genome. **sense **– the fraction of matches in the sense orientation of known or hypothetical transcription units. **antisense **– the fraction of matches in the antisense orientation of known or hypothetical transcription units.

The cloning strategy was designed to preserve the orientation of the cDNAs. The first strand directional random primer, DRP1, contained, from 5' to 3', 12 nt of defined buffer sequence, the 8 nt *Asc*I restriction endonuclease sequence, 5 nt of random sequence and a 3' T nucleotide (Fig. 1A). The 5' buffer sequence was included to prevent destruction of the *Asc*I site by the 5'->3' exonuclease activity of *E. coli Pol*I during second-strand synthesis. Using a primer which did not contain the buffer sequence resulted in the frequent appearance of cDNA clones that had lost the *Asc*I site and therefore orientation (data not shown). T was added to the 3' end and As were excluded from the defined primer sequence at the cost of initiating cDNA synthesis at A (Fig. 1A-1, 2, 4) to eliminate an observed DRP1 self-priming artifact during first-strand synthesis.

We tested the procedure by sequencing random clones picked from the different cDNA libraries. In an initial test, 43 clones from the Amphioxus library, 54 from the zebrafish library, and 15 from the *Drosophila *library were sequenced. Of the 112 clones, 9 clones contained no cDNAs and 4 clones yielded unreadable sequence data. The remaining sequences were compared to the NCBI non-redundant combined protein, combined DNA, and combined EST sequence databases, using the TBlastX search procedure [20]. In the self-subtracted libraries, 59 sequences matched translated RNAs, ESTs, or gene exons (e < 10^-5^) and 87% of these were in the forward reading frame. This fraction is within the 80–95% observed in directional cDNA libraries made by standard approaches using oligo dT primed cDNAs [21], indicating that the directional cloning strategy functions properly. that cloning was, as designed, directional. 16 matches to genomic DNA that were not part of any known functional or encoding RNA were identified. Since the first strand priming technique was not completely random and the directional primer included additional 5' GC-rich sequence (Fig. 1, parts 1,2), we were concerned that priming might be biased towards GC-rich regions. We examined this using clones which identified perfect matches in the sequence databases. From the 55 such clones we collected the 1100 nucleotides of sequence data lying within 20 nt of the 3' end of the random primer, i.e. under the non-random 5' portion of DRP1. These sequences were 49% G+C, indicating that the additional GC rich sequence in the 5' portion of the first strand cDNA primer did not seriously bias the priming process. 74% of the sequenced clones in the self-subtracted libraries contained an A at the sixth nucleotide following the *Asc*I restriction site in the primer, indicating that cDNA synthesis mostly initiated, as designed, at an A (Fig. 1A).

Since these data provided an initial indication that the library construction technique was working as expected, we randomly picked and sequenced another 200 colonies from the *Danio rerio *library. All the *Danio rerio *and *Drosophila *sequences were then compared to the NCBI non-redundant nucleotide sequence database (GenBank+EMBL+DDBJ+PDB sequences – but no EST, STS, GSS, environmental samples or phase 0, 1 or 2 HTGS sequences) using TBLASTX and to the Ensembl unmasked zebrafish genome (Zv6, Ensemble genebuild, Aug. 2006) using BLASTN set to oligo sensitivity. Of the 257 sequences, 10 sequences showed inconclusive or no matches. The remainder matched genomic DNA to better than 97%, indicating that the genomic source contexts of almost all cDNAs were identified (see Additional file 1). These were then categorized with respect to known or hypothetical transcription units (Fig. 2). 245 of the 247 matches are unique in sequenced population, coming from different loci. Although the source material for these libraries was partially degraded total RNA, 70% of the sequences matched known or hypothetical mRNAs. 17% of these were novel, being either first matches to hypothetical mRNA sequence or unknown splice variants of previously identified mRNA transcription loci. 16% of sequences matched hnRNA sequence likely removed during mRNA splicing – which does not necessarily mean that they are non-functional. Eight matches to known or hypothetical ncRNA loci were identified. These included one match to a large rRNA transcription unit and 5 matches to known or hypothetical ncRNA transcription units encoding miRNAs. A total of 17 matches were outside either known or predicted transcription units. One of these exhibited discontinuous matches to very closely linked same-strand sequence in the genome, indicative of splicing, and suggested a novel unpredicted transcription unit. A total of 10 clones, some in known or predicted introns and others outside of predicted transcription units exhibited significant (e < 10^-9^) matches to a large number of loci (>50) dispersed throughout the genome. These may represent undescribed interspersed repeat elements (LINES and SINES) in the zebrafish.

In 227 of the matches, orientation of the cDNAs could be assigned relative to known or hypothetical loci. Of these, 80% were in the sense orientation. Of the antisense matches, only four could clearly be considered artifacts of cloning. 13 antisense clones were structurally normal, exhibiting the appropriate transitions from cDNA to vector sequences, but were spliced according to their sense strand match. Although this suggests that the antisense orientation is an artifact of cloning, such precisely matching non-canonical splicing has been reported for at least one genuine antisense transcript [22].

We turn finally to the question of the extent of subtraction. The library data clearly show that the cDNA population was subtracted enough to remove most representation of the abundant rRNAs and greatly increase the representation of the different mRNA species. However, equalization was not complete. Since the transcriptome contains all intron sequences, which greatly exceed the mRNA population in complexity (at least in the zebrafish), a complete subtraction would be expected to contain far more intron than mRNA sequence. This is not the case in the libraries examined, where mRNA sequences outnumber intron sequences by a ratio of almost 7:1. We addressed the issue of equalization within the mRNA population in two different ways. We determined the frequency of β-actin sequence, a particularly abundant mRNA, by probing the 200–300 bp zebrafish library with antisense oligonucleotide. Only one β-actin clone was identified among 5 × 10^4 ^colonies. Since β-actin represents approximately 1% of the mRNAs of embryonic zebrafish heart [23] and is relatively constant across tissues under physiological conditions [24], the representation of this abundant mRNA has been reduced in the equalized libraries. In the second approach, we compared mRNA representation among our sequences with mRNA representation in an unsubtracted cDNA library constructed from 72 hour embryonic zebrafish heart [23]. In the heart library, there are 11 mRNAs that are represented at frequencies between 0.4% (the ADP/ATP carrier protein) and 4.3% (the ribosomal proteins). Of these abundant mRNAs, two are represented once in our collection of 240 zebrafish cDNA sequences and the rest were not represented at all. There were two other mRNA loci that were represented twice among the zebrafish self-subtracted sequences, one encoding Ankyrin 3 and the other encoding Ran-binding protein 2. Neither of these two loci were represented in the 5000 sequences from the heart library.

There are several points of caution in using the procedure. In self-subtraction, it is the complexity of contaminating sequence, rather than its abundance, that may determine the extent of contamination in the final library. Even a few picograms of genomic DNA has a greater complexity than the entire functional RNA population. Although the importance and extent of non-coding transcripts is under revision [25] we did not observe any advantage to increasing the extent of self subtraction, either by including additional rounds of self subtraction or by increasing the C_o_t value of the subtractions. Clones chosen from a fourth library that had gone through an additional round of self-subtraction and amplification did not match any mRNAs (data not shown.)

The goal of the described procedure was to have as much as possible of the entire RNA population represented in a library, not to have it represented in full length copies. Some functional non-coding RNAs are almost certainly excluded from the libraries. We have used the procedure successfully on cDNA fragments as small as 100–200 bp. Self subtracted libraries of smaller sequences may be possible if the phosphate concentration in the hydroxylapatite binding buffer is decreased and only small sequences are included in the reaction. The smallest functional processed RNAs, e.g. the 22 nt microRNAs, will not be represented, although their primary transcripts certainly are. Since the procedure functions via inter-molecular reassociation kinetics, sequences with extensive self-homology (snap-back) will be removed independent of their abundance in the RNA population. The procedure will not work well for most full length transcripts since amplifying sequences larger than even 500 base-pairs in size is more difficult and more sensitive to reaction conditions. Even in our target size range of 200–300 bp there are undoubtedly some sequences that do not amplify well by PCR and are therefore underrepresented. We believe that the more complete representation possible with short sequences offsets the disadvantage of not cloning full length copies, especially as the sequences are more than long enough to identify significant matches in the databases, encode many complete protein domains, and to serve as probes for cloning or assembling full-length cDNAs by the many other methods available.

## Conclusion

The simple procedures described here permit the construction of high-quality, directional cDNA libraries using small amounts of degraded total RNA. Since the method does not distinguish between polyA^+ ^and polyA^- ^species, all RNAs above 100 nt may be represented, including polyA^- ^mRNAs and many functional but non-translated RNAs. The procedure should prove valuable in situations where more complete representation of the transcriptome is desired.

## Methods

The following procedural details are relevant to the successful use of the method. They are cited in the Results and Discussion text as (Methods-#).

(1) The removal of genomic DNA contamination and purity of sample at the RNA extraction step is crucial. The self-subtraction procedure is more sensitive to the complexity of a contaminant than its abundance. Tissues should be processed in either disposable plasticware or baked glassware.

(2) Partial hydrolysis of the RNA is an important step. The method is PCR-based and PCR efficiency becomes increasingly sequence and reaction-condition dependent as the size of the template increases. To avoid this source of bias, short cDNAs are used. Producing these by shortening the RNA template through random hydrolysis has the added advantage of reducing the formation of intramolecular secondary structure that can interfere with priming and reducing the expected bias for the 5' ends of full-length molecules.

(3) The quality of the cDNA syntheses and subsequent PCR amplification steps was evaluated by tracing the reactions with ^32^P-dCTP and then determining the incorporated fraction to estimate the yield and agarose gel electrophoresis to gauge the quality of the reaction.

Oligonucleotide quality was found to be important. We used HPLC grade oligonucleotides for the library construction and self-subtraction. DRP1 is 5'P-GCTCGCCCTCGCGGCGCGCCNNNNNT. The lone-linker LL1 is the annealed product of LL1F and LL1R

5'CTGGCTCGCCCTCGCGGATCCG (LL1F)

AGACCGAGCGGGAGCGCCTAGGC 5' (LL1R)

(4) LL1P is 5'CTGGCTCGCCCTCGCGG. The correct amount of template and the number of cycles were determined empirically. Using the reaction conditions described, a yield of between 1 and 5 μg was typical. A yield greater than this may be stressing the reaction, resulting in partial reaction products and other artifacts.

(5) The hydroxylapatite was de-fined prior to use by resuspending the powder in a large volume of annealing buffer, allowing the matrix to settle by gravity, and removing the still slightly cloudy upper phase. This was repeated 3–5 times. 1 ml of hydrated hydroxylapatite binds approximately 100 μg of DNA.

(6) Failure to completely remove all hydroxylapatite results in binding of ssDNA as the phosphate concentration drops during the next buffer exchange step.

(7) Depending upon the extent of annealing, the concentration of remaining ssDNA may be low enough to result in a significant fraction binding to the filter membrane. To prevent this, the centricons should be passivated in 5% Tween-20 for one hour followed by four rinses with ddH_2_O.

## Authors' contributions

CD made the libraries and assisted in writing the paper. ZB assisted in making the libraries and completed the analysis. CD and ZB contributed equally to this work. IG participated in the design of the study, analysis of results, and writing the paper.

## Supplementary Material

Additional file 1 The supplementary data file lists properties of cDNA sequences that match genomic loci in the sequence databases. Click here for file
