# Supplementary material for: A method for the construction of equalized directional cDNA libraries from hydrolyzed total RNA
Source: BMC Genomics. 2007 Oct 9;8:363. doi: 10.1186/1471-2164-8-363 (PMC2134933; doi:10.1186/1471-2164-8-363)
Supplement: Additional file 1 — The supplementary data file lists properties of cDNA sequences that match genomic loci in the sequence databases. [file 1471-2164-8-363-S1.pdf]

Supplementary material for (Davis et al., A method for the construction of equalized directional cDNA libraries from hydrolyzed total RNA)

Table A1. Database matches to *Danio rerio* clones from the self-subtracted libraries. All clones for which a source locus in the genome (>97% identity) could be identified are listed. **clone ID** - A unique identifier for the query sequence from the cDNAs. DrXXX are from the 48 hour embryonic *Danio rerio* 200-300 bp self-subtracted cDNA library. Accession - EMBL accession number of clone. **cDNA size (bp)** - shows the size of the cDNA clones in base-pairs. If sequence quality deteriorated before the entire cDNA was sequenced, then the size is indicated with a > to show the length of high quality sequence present. **Clone integral** - "Yes" indicates that vector sequence spliced onto insert sequence through the expected linker sequence and that there were no internal cloning endonuclease sites. **Match locus description** - indicates the nature of the matching sequence in the sequence databases. **Match locus ID** - Where possible the Ensembl gene name for any matches is given. **hit type** - Each match was catagorized as one or a combination of the following: mRNA, hnRNA (intron), ncRNA (non-coding RNA), NonTrans - not known or predicted to be translated. **known/ Hypothetical** - If the match was to sequence that had already been identified as transcribed it as marked as known, if the sequence was part of a predicted transcription locus based on either the GeneSnap or GeneScan programs, but was not yet supported by cDNA data, then it was marked as hypothetical.

clone ID	Accession	cDNA size (bp)	clone integral	Match locus description	Match locus ID	hit type	known/Hypothetica
Dr001	AM849824	228	Yes	Danio rerio fast muscle specific-myosin heavy chain mRNA	gi|14794383|gb|AF165817.1|AF	mRNA	known
Dr002	AM849869	198	Yes	Chr:20, in EST intron, but also partial sequence homology to XI imitation switch exon.	ENSDARESTG00000018506, GE	mRNA, hnRNA	known, Hypothetica
Dr003	AM849868	>188	No	Chr:10, inside a hypothetical intron, but perfect match to EST from mRNA	GENSCAN00000002979, CT658	mRNA	known
Dr004	AM849867	240	Yes	MYOSIN HEAVY CHAIN	ENSDARG00000001993	mRNA	known
Dr005	AM849866	296	No	AAA+ ATPase, core, ATP Binding Cassette transporter mRNA	ENSDARG00000062661	mRNA	known
Dr006	AM849865	249	Yes	Rabbit sperm membrane protein-B (RSD-1)	ENSDARG00000063710, gi|194	mRNA	known
Dr007	AM849864	253	Yes	Chr:13 outside anything known, but inside predicted intron	GENSCAN00000038321	hnRNA	Hypothetical
Dr008	AM849863	>192	No	Chr:2 intron sequence, just past exon of known gene	ENSDARG00000021739, zgc: 1	hnRNA	known
Dr009	AM849862	191	Yes	Chr:14 in intron right at boundary with likely exon	ENSDARESTG00000012252	hnRNA	known
Dr010	AM849861	194	Yes	**Chr:15 in intron of zgc:63812**	ENSDARG00000032614, gi|834	hnRNA	known
Dr011	AM849860	194	Yes	Fatso homolog,ZFIN_ID: fto	ENSDARG00000044216, ZFIN_I	mRNA	known
Dr012	AM849859	254	Yes	hCDC10=CDC10 homolog	ENSDARG00000052673, gi|560	mRNA	known
Dr013	AM849858	225	Yes	Chr:21 in a 5'UTR of assembled EST contig	ENSDARESTG00000011428,	mRNA	known
Dr014	AM849857	230	Yes	similar to Nebulin (LOC566928)	ENSDARG00000032630, ZFIN_I	mRNA	known
Dr015	AM849856	276	Yes	3' of Zebrafish Cullin Vasopressin-activated calcium-mobilizing receptor (VACM-1)	gi|47086626|ref|NM_212706.1|	NonTrans	known
Dr016	AM849855	>186	No	Genome: Ch13, intron of known transcript	ENSDARESTT00000000626	hnRNA	known
Dr017	AM849854	214	Yes	Genome: Chr:1 in an intron, homology is split by small region	ENSDARG00000060321, LOC79	hnRNA	known
Dr018	AM849853	198	Yes	Matchs three hypothetical coding transcripts on Ch20, 22, and 5	GENESCAN00000035374	mRNA	Hypothetical
Dr019	AM849852	233	Yes	Match on Ch19 similar to NUP196 nucleoporin	ENSDARESTT00000033290	mRNA	known
Dr020	AM849823	236	Yes	Matches an EST intron	ENSDARESTG00000022656	hnRNA	known
Dr021	AM849851	234	Yes	Genome: Ch23, matches intron of fructose 6-phosphate,2-kinase	ENSDARG00000023840	hnRNA	known
Dr022	AM849850	275	Yes	transketolase variant	ENSDARG00000029689, gi|146	mRNA	known
Dr023	AM849849	198	Yes	Danio rerio mRNA for tenascin-W	ENSDARG00000024829, gi|276	mRNA	known
Dr024	AM849848	289	Yes	A kinase (PRKA) anchor protein 10	ENSDARG00000059549, gi|991	mRNA	known
Dr025	AM849847	203	Yes	arginine N-methyltransferase p77	ENSDARG00000051902 ,prmt7	mRNA	known
Dr026	AM849846	278	Yes	chromosome segregation 1-like gene="Cse1l", Mus musculus cellular apoptosis susceptibility protein mRNA	ENSDARESTG00000000723	mRNA	known
Dr027	AM849845	113	Yes	protein kinase, cGMP-dependent, type I (prkg1), 5'UTR	ENSDARG00000020656, ZFIN_I	mRNA	known
Dr028	AM849844	252	No	ENSDARESTP00000016788	ENSDARESTG00000010216	mRNA	known, Hypothetica
Dr029	AM849843	325	Yes	RuvB-like 1 (zPontin)	ENSDARG00000002591	mRNA	known
Dr030	AM849842	253	Yes	transcriptional co-repressor SIN3A	ENSDARG00000059812	mRNA	known
Dr031	AM849841	310	Yes	Match on Ch5 outside any transcript, but in big Ab initio introns, both orientations	GENESCAN00000018153	hnRNA	Hypothetical
Dr032	AM849840	254	Yes	KIAA1199 protein, colon cancer secreted protein 1, membrane protein 2	ENSDARG00000030427	mRNA	known
Dr033	AM849839	233	Yes	Matches an Ab initio Genscan mRNA, similarity to an endonuclease	GENSCAN00000009827	mRNA	known, Hypothetica
Dr034	AM849838	162	No	Danio rerio KIAA0650-like protein mRNA	ENSDARG00000014836, ZFIN_I	mRNA	known
Dr035	AM849837	236	Yes	CRM1/XPO1 protein mRNA exportin 1 homolog	ENSDARG00000057200, ZFIN_I	mRNA	known
Dr036	AM849836	240	Yes	similar to procollagen, type XXVII, alpha 1	ENSDARG00000034373	mRNA	known
Dr037	AM849835	201	Yes	EST gene: Danio rerio similar to C2-HC type zinc finger protein X-MyT1	ENSDARESTG00000001656	mRNA	known
Dr038	AM849834	286	Yes	Danio rerio similar to alpha 5 type IV collagen isoform 2, precursor, ab initio only	FGENESH00000056858	mRNA	known, Hypothetica
Dr039	AM849833	186	Yes	Chr:9 intron of interleukin 10 receptor 2 gene	ENSDARG00000004385, ZFIN_I	hnRNA	known
Dr040	AM849832	218	Yes	Danio rerio similar to mouse a1(XI) collagen	GENSCAN00000006384, gi|684	mRNA	Hypothetical
Dr041	AM849831	271	Yes	Danio rerio CDC5	ENSDARG00000043797, gi|174	mRNA	known
Dr042	AM849830	223	Yes	Danio rerio mitochondrion, homologous to cytochrome C oxidase subunit III mRNA	gi|15079186|ref|NC_002333.2|	mRNA	known
Dr043	AM849829	253	No	Glycoside hydrolase, family 2 mRNA	ENSDARG00000063126	mRNA	known
Dr044	AM849828	216	Yes	Danio rerio similar to branched chain keto acid dehydrogenase E1	ENSDARG00000040555	mRNA	known
Dr045	AM849827	202	Yes	Genome: Chr:17, outside of anything known, but in ab initio intron	GENSCAN00000041222, gi|737	hnRNA	Hypothetical
Dr046	AM849826	234	Yes	Danio rerio similar to CG33456-PA	ENSDARESTG00000022973	mRNA	known
Dr047	AM849825	180	Yes	Chr:3	ENSDARESTG00000032754, gi|	hnRNA	known
Dr048	AM849947	279	Yes	Danio rerio zgc:65870 (zgc:65870), mRNA	ENSDARG00000024276, NM_21	mRNA	known
Dr049	AM849946	256	Yes	Ensemble protein: LOC558831, Gallus gallus SOUL protein (SOUL), mRNA	ENSDARG00000061881, NM_2(	mRNA	known
Dr050	AM849945	229	Yes	Apolipoprotein related	ENSDARESTG00000024305	mRNA	known
Dr051	AM849944	253	Yes	Danio rerio actin, alpha, cardiac muscle 1 (actc1), mRNA	ENSDARG00000055618, NM_21	mRNA	known
Dr052	AM849943	224	Yes	Danio rerio EST transcript TOM1 TARGET OF MYB	ENSDARESTG00000003853	mRNA	known
Dr053	AM849942	230	Yes	Chr:2 outside any known or hypothesized transcription unit	GENESCAN00000014245	hnRNA	Hypothetical
Dr054	AM849941	256	Yes	Danio rerio similar to MGC81123 (O-acyltransferase (membrane bound)	GENSCAN00000019802, XM_0(	mRNA	known, Hypothetica
Dr055	AM849940	195	Yes	PREDICTED: hypothetical protein [Danio rerio]	ENSDARG00000060238, XP_69	mRNA	known, Hypothetica
Dr056	AM849939	180	Yes	PREDICTED: Danio rerio similar to echinoderm microtubule associated protein like 5 (LOC570011), mRNA	ENSDARG00000060403, XM_69	mRNA	known
Dr057	AM849938	190	Yes	Danio rerio alpha II-spectrin mRNA	ENSDARG00000060403, EF375	mRNA	known
Dr058	AM849937	215	Yes	Danio rerio cDNA clone MGC:162583, similar to Gelsolin precursor	ENSDARG00000045262, BC135	mRNA	known
Dr059	AM849936	201	Yes	Chr:22 in an intron of known gene, nuclear factor I/A	ENSDARG00000062420, zgc:15	hnRNA	known
Dr060	AM849935	209	Yes	PREDICTED: Danio rerio similar to cardiac muscle tensin	GENSCAN00000021848, XM_0(	hnRNA	Hypothetical
Dr061	AM849934	234	Yes	Chr:3, antisense to Ab initio genescan unit	GENESCAN00000036643	mRNA	Hypothetical
Dr062	AM849933	209	Yes	Danio rerio SEC14-like 1 (S. cerevisiae) (sec14l1), mRNA	ENSDARG00000019301, NM_2(	mRNA	known
Dr063	AM849932	259	Yes	PREDICTED: Danio rerio similar to ankyrin 3 (LOC571429)	ENSDARG00000063107, XM_69	mRNA	known
Dr064	AM849931	267	Yes	Chr:3 inside intron ensemble known coding gene	ENSDARG00000033638, LOC79	hnRNA	known
Dr065	AM849930	287	Yes	Chr:16, outside any known or hypothesized transcription unit		NonTrans	
Dr066	AM849929	184	Yes	ncRNA locus containing miRNA	ENSDART00000093491, BC135	ncRNA, hnRNA	Hypothetical
Dr067	AM849928	244	Yes	Chr12 outside any known or hypothesized transcription unit		NonTrans	
Dr068	AM849927	248	Yes	Danio rerio zgc:153593, mRNA, apolipoprotein D similarity	ENSDARG00000060345, INP_0(	mRNA	known
Dr069	AM849926	230	Yes	PREDICTED: Danio rerio hypothetical protein LOC100001267, microtubule associated serine/threonine kinas	ENSDARG00000059508, XM_0(	mRNA	known
Dr070	AM849925	199	Yes	Chr:4 inside intron of zgc:92710, a tight junction component.	ENSDARG00000006747, zgc:92	hnRNA	known
Dr071	AM849924	230	Yes	matches multiple miRNA loci in the genome.	ENSDARG00000063790	ncRNA, hnRNA	known, Hypothetica
Dr072	AM849923	253	Yes	Danio rerio stromal cell-derived factor 2 (sdf2), mRNA	ENSDARG00000054857, XM_0(	mRNA	known
Dr073	AM849922	209	Yes	Danio rerio zgc:77282, mRNA, LIM-kinase1 similar	ENSDARG00000042252, BC056	mRNA, hnRNA	known
Dr074	AM849921	214	Yes	PREDICTED: Danio rerio similar to mKIAA1585 protein	GENSCAN00000004279, XM_0(	mRNA	Hypothetical
Dr075	AM849920	205	No	Chr:2, inside intron of zgc:114073, TRANSLOCATION protein 1, SEC62 homolog	ENSDARG00000019951, tbc1	hnRNA	known
Dr076	AM849919	214	Yes	Chr:18, inside intron of insulin-like growth factor1 receptor like	ENSDARG00000027423, igf1ra	hnRNA	known
Dr077	AM849918	243	Yes	Danio rerio spondin 1b, mRNA	ENSDARG00000023694, NM_1	mRNA	known
Dr078	AM849917	252	Yes	Danio rerio NeuroD (nrd) gene, complete cds	ENSDARG00000019566	mRNA	known
Dr079	AM849916	234	Yes	Danio rerio si:ch211-219i10.1, mRNA, similar to vertebrate apolipoprotein B	ENSDARG00000022267, NM_0(	mRNA	known
Dr080	AM849915	295	Yes	Chr:7,ncRNA transcription unit containing miRNAs.	ENSDARG00000063790	ncRNA, hnRNA	Hypothetical
Dr081	AM849914	235	Yes	Danio rerio zgc:158428, mRNA, similar to human and mouse ubiquitin specific peptidase 39	ENSDARG00000041908, NM_0(	mRNA	known
Dr082	AM849913	268	Yes	chromodomain helicase DNA binding protein 4, Danio rerio cDNA clone IMAGE:7137214, partial cds	ENSDARESTG00000014587, BC	mRNA	known
Dr083	AM849912	187	Yes	transportin 2 (importin 3, karyopherin beta 2b) [Danio rerio]	ENSDARG00000038239, NP_00	mRNA	known
Dr084	AM849911	231	Yes	Chr:4, outside any known or predicted transcription unit		NonTrans	
Dr085	AM849910	240	Yes	mixed: Danio rerio hypothetical LOC559226 (LOC559226), mRNA - similar to trans-Golgi p230	GENSCAN00000045595, XR_02	mRNA	known, Hypothetica
Dr086	AM849909	230	Yes	Chr:7, antisense to intron of ENSDARESTG00000013027, a very small gene based on EST, coding ~ 50 aa	ENSDARESTG00000013027	hnRNA	known, Hypothetica
Dr087	AM849908	256	Yes	PREDICTED: Danio rerio hypothetical LOC564934 (LOC564934), mRNA, similar to zinc finger E-box binding	ENSDARG00000059564, XM_68	mRNA	known
Dr088	AM849907	203	Yes	Chr:14, outside of any known or predicted transcription unit		NonTrans	
Dr089	AM849906	199	Yes	Danio rerio lactate dehydrogenase A4, mRNA	ENSDARG00000040856, BC045	mRNA	known
Dr090	AM849905	217	Yes	Danio rerio zgc:91987growth factor receptor-bound protein 10	ENSDARG00000002013, NM_0(	mRNA	known
Dr091	AM849904	238	Yes	Danio rerio clone RK073A1F03 cell division cycle 42	ENSDARG00000044573, AY398	mRNA	known
Dr092	AM849903	224	Yes	matches established duplicate genes, antisense in 3'UTR in both, one is proteosome subunit beta	ENSDARG00000053697, ZFIN_I	mRNA	known
Dr093	AM849902	214	Yes	Danio rerio laminin receptor 1 (LAMR1, rpsa) mRNA	ENSDARG00000019181	mRNA	known
Dr094	AM849901	302	Yes	Chromosome segregation 1-like homology, mRNA	ENSDARESTG00000016410, BC	mRNA	known
Dr095	AM849900	>100	No	RefSeq_dna: XR_029557.1, similar to Multiple EGF-like-domains 10 (LOC564571)	ENSDARG00000017229	mRNA	known
Dr096	AM849899	249	Yes	Danio rerio zgc:56559, mRNA	ENSDARG00000035838, BC071	mRNA	known
Dr097	AM849898	219	Yes	SWI/SNF related, matrix associated, actin dependent regulator of chromatin, subfamily e, member 1	ENSDARG00000016871, BC066	mRNA	known
Dr098	AM849897	215	Yes	Chr:21, inside intron of a cyclin gene	ENSDARG00000061693	hnRNA	known
Dr099	AM849896	217	Yes	col5a2l , simlilar to collagen,	ENSDARG00000031678, col5a2	mRNA, hnRNA	known
Dr100	AM849895	256	Yes	Danio rerio flotillin 2a,	ENSDARG00000004830, ZFIN_I	mRNA	known
Dr101	AM849894	188	No	Ch14, Antisense inside an ab initio intron of a very tiny gene, coding for 89 aa	FGENESH00000065374	hnRNA	Hypothetical
Dr102	AM849893	190	Yes	Chr:10, outside any known transcription unit, but forward orientation inside an abinitio intron extension of E	GENSCAN00000042632	hnRNA	Hypothetical
Dr103	AM849892	248	Yes	Chr:1, inside intron of ab initio extension of a known gene	GENSCAN00000014679	hnRNA	Hypothetical
Dr104	AM849891	255	Yes	extension of known transcription unit ENSDARESTG00000017154	GENSCAN00000029957	mRNA	known, Hypothetica
Dr105	AM849890	219	Yes	PREDICTED: Danio rerio hypothetical LOC565428, mRNA, myomesin similar	ENSDARG00000007165	mRNA	known
Dr106	AM849889	260	Yes	dynein cytoplasmic 1 intermediate chain 2	ENSDARG00000005756	mRNA	known
Dr107	AM849888	238	Yes	shaker-like voltage gated potassium channel	ENSDARG00000060508, XM_68	mRNA	known
Dr108	AM849887	95	Yes	Inside an exon of an EST gene, not similar to anything	ENSDARESTG00000028721	mRNA	known

Dr109	AM849886	252	Yes	PROSTAGLANDIN F2 RECEPTOR NEGATIVE REGULATOR	ENSDARG00000063659	mRNA	known
Dr110	AM849885	>223	No	Melanin-concentrating hormone 1 receptor	ENSDARG00000062332	mRNA	known
Dr111	AM849884	243	Yes	Chr:7, outside anything known, but inside an abintio gene scan intron	GENESCAN00000038859	hnRNA	Hypothetical
Dr112	AM849883	216	Yes	inositol hexaphosphate kinase 2 (ihpk2), intron	ENSDARG00000008310, wu:fb4	hnRNA	known
Dr113	AM849882	198	Yes	Danio rerio kelch-like 20 (Drosophila)	ENSDARG00000038801, NM_21	mRNA	known
Dr114	AM849881	248	Yes	Danio rerio tumor necrosis factor, alpha-induced protein 1 - tnfaip1	ENSDARG00000041565, NM_1	mRNA	known
Dr115	AM849880	215	Yes	Ran-binding protein 2	ENSDARG00000063710, XM_0	mRNA	known
Dr116	AM849879	238	Yes	overlaps Danio rerio similar to Apoptotic chromatin condensation inducer in the nucleus	GENSCAN00000027330, XM_0	mRNA	Hypothetical
Dr117	AM849878	235	Yes	similar to ATP-binding cassette transporter 13 (LOC559093), mRNA	ENSDARESTG00000010801, XM	mRNA	known
Dr118	AM849877	214	Yes	Chr:1, spans predicted intron/exon boundary, overlaps a short Neurobeachin like sequence	GENSCAN00000022131, ENSDA	mRNA, hnRNA	known, Hypothetica
Dr119	AM849876	229	Yes	Danio rerio LOC567461	ENSDARG00000061490	mRNA	known
Dr120	AM849875	238	Yes	Peptidase S1A, chymotrypsin	ENSDARG00000061830, GENSC	hnRNA	known, Hypothetica
Dr121	AM849874	242	Yes	Chr:24, Not contained in any one predicted transcription unit - spans two, but does not match predicted exons		mRNA	Novel, Hypothetical
Dr122	AM849873	203	Yes	PREDICTED: Danio rerio wu:fi75b02, transcript variant 1, cyclin T1	ENSDARG00000017525, wu:fi7	mRNA	known
Dr123	AM849872	201	Yes	Chr:16, spans intron-exon boundary of what is probably a Bicaudal-C homolog	GENSCAN000000003396	mRNA, hnRNA	known, Hypothetica
Dr124	AM849871	257	Yes	Chr:15 3 equal matches	ENSDARG00000061595	NonTrans	
Dr125	AM849870	230	Yes	Danio rerio neurobeachin (nbea), mRNA	ENSDARG00000010158	mRNA	known
Dr126	AM849985	203	Yes	Danio rerio bone morphogenetic protein 1a	ENSDARG00000028071, NM_0	mRNA	known
Dr127	AM849984	230	Yes	Danio rerio matrix metalloproteinase 2	ENSDARG00000017676, BC076	mRNA	known
Dr128	AM849983	234	Yes	Danio rerio protein inhibitor of activated STAT, 2,	ENSDARG00000059831, BC068	mRNA	known
Dr129	AM849982	257	Yes	Similar to semaphorin 6D	GENSCAN00000025421, XM_0	mRNA	Hypothetical
Dr130	AM849981	241	Yes	Chr:25, extension of GENESCAN00000034583		NonTrans	
Dr131	AM849980	193	Yes	Danio rerio activin receptor IIA mRNA	ENSDARG00000011188, AY962	mRNA	known
Dr132	AM849979	255	Yes	PREDICTED: Danio rerio hypothetical protein LOC791902	ENSDARG00000037642, XM_0	mRNA	known
Dr133	AM849978	236	Yes	PROBABLE TUMOR SUPPRESSOR MN1	ENSDARG00000060281, XM_6	mRNA	known
Dr134	AM849977	238	Yes	Danio rerio milk fat globule-EGF factor 8 protein	ENSDARG00000045803, NM_0	mRNA	known
Dr135	AM849976	>164	No	cationic amino acid transporter	ENSDARG00000051887	mRNA	known
Dr136	AM849975	248	Yes	No chromosomal location, Contig Zv6_NA307.		NonTrans	
Dr137	AM849974	234	Yes	Danio rerio cDNA clone MGC:163029, homolog of dipeptidyl peptidase III	ENSDARG00000061068, BC139	mRNA	known
Dr138	AM849973	274	Yes	PREDICTED: Danio rerio hypothetical protein LOC794477	ENSDARESTG00000009909, XM	mRNA	known
Dr139	AM849972	193	Yes	Chr:4, adjacent to microRNA dre-mir-739, sequence pieces conserved across metazoans	dre-mir-739	ncRNA, hnRNA	known
Dr140	AM849971	227	Yes	zgc: 152785, similarity of nucleolar phosphoprotein, an undescribed splice variant	ENSDARG00000004636, Dzgc:	mRNA	known
Dr141	AM849970	220	Yes	Danio rerio protein kinase C, novel (prkch)	ENSDARG00000043243, NM_0	mRNA	known
Dr142	AM849969	>132	No	Similar to Goldfish growth-associated protein (GAP-43)	ENSDARG00000063725, M262	mRNA	known
Dr143	AM849968	200	Yes	ZFIN_ID zgc:64095	ENSDARG00000045422, XM_0	mRNA	known
Dr144	AM849967	73	No	Ch23, outside any known or predicted transcription unit		NonTrans	
Dr145	AM849966	211	Yes	PREDICTED: Danio rerio similar to heparan sulfate 2-O-sulfotransferase	ENSDARG00000062008, XM_6	mRNA	known
Dr146	AM849965	169	Yes	Ch15, outside of any known/predicted coding region		NonTrans	
Dr147	AM849964	216	Yes	Danio rerio integrin, beta 1b (ZFIN_ID: itgb1b)	ENSDARG00000053255, NM_0	mRNA	known
Dr148	AM849963	234	Yes	Danio rerio similar to ankyrin 3 (LOC571429)	ENSDARG00000063107, XM_6	mRNA	known
Dr149	AM849962	199	Yes	MYOSIN REGULATORY LIGHT CHAIN, Danio rerio zgc:103639 mRNA...	ENSDARG00000002589, BC081	mRNA	known
Dr150	AM849961	196	Yes	Chr:9, antisense in intron of EST gene	ENSDARESTG00000011550	hnRNA	known
Dr151	AM849960	206	Yes	titin-like (ttnl), mRNA	ENSDARG00000028213, XM_67	mRNA	known
Dr152	AM849959	183	Yes	Danio rerio LOC569156, ZFIN_ID: si:dkey-261h15.1, Protein kinase	ENSDARG00000062082, XM_6	mRNA	known
Dr153	AM849958	185	No	PREDICTED: Danio rerio similar to Phosphatidic acid phosphatase type 2B (LOC557680), mRNA	GENSCAN00000026589, ENSDA	mRNA	known, Hypothetica
Dr154	AM849957	194	Yes	Danio rerio LOC572155, similar to Deltex protein 1, Zinc finger, RING-type	ENSDARG00000062840, XM_6	mRNA	known
Dr155	AM849956	235	Yes	similar to diaphanous homolog 2 (Drosophila), splicing from two hypothetical exons of different genes in opp	GENESCAN00000036506, GENE	mRNA	Hypothetical
Dr156	AM849955	236	Yes	Danio rerio phospholipase A2-activating protein (plaa), mRNA	ENSDARG00000042728, NM_21	mRNA	known
Dr157	AM849954	266	Yes	Chr:10, outside of any known or predicted transcription unit		NonTrans	
Dr158	AM849953	206	Yes	Chr:3, just outside tweety coding region, in 5' UTR of its gene	ENSDARG00000059650	mRNA	known
Dr159	AM849952	236	Yes	Danio rerio tyrosyl-tRNA synthetase (yars), mRNA	ENSDARG00000035913, NM_2	mRNA	known
Dr160	AM849951	>71	No	Piece of collagen type II, alpha-1 at least		mRNA	
Dr161	AM849950	271	Yes	Danio rerio cDNA clone MGC:163098 IMAGE:8106216, complete cds, similar to U2-associated SR140 protei	ENSDARG00000060232, BC135	mRNA	known
Dr162	AM849949	219	Yes	PREDICTED: Danio rerio similar to protocadherin1-alpha-av3-vCP (LOC100000793), mRNA	GENSCAN00000028382, XR_02	mRNA	Hypothetical
Dr163	AM849948	238	Yes	PREDICTED: Danio rerio hypothetical LOC555685 (LOC555685), GNOMON, supported by EST evidence	XM_698072.2	NonTrans	
Dr164	AM850018	>166	No	Danio rerio transmembrane protein 30A, mRNA	ENSDARG00000030236, BC074	mRNA	known
Dr165	AM850017	300	Yes	Danio rerio heat shock protein 9 (hspa9), mRNA	ENSDARG00000003035, NM_2	mRNA	known
Dr166	AM850016	240	Yes	PREDICTED: Danio rerio hypothetical protein LOC100001684, similar to titin (TTN)	ENSDARG00000000563, XM_0	mRNA	known
Dr167	AM850015	246	Yes	Danio rerio Na+/K+ ATPase alpha2 subunit	ENSDARG00000010472, AY028	mRNA	known
Dr168	AM850014	246	Yes	Danio rerio similar to brain-specific angiogenesis, inhibitor 3 (LOC566117), mRNA	ENSDARG00000059819, XM_6	mRNA	known
Dr169	AM850013	292	Yes	PREDICTED: Danio rerio hypothetical protein LOC100003103	ENSDARESTG00000026396, XM	mRNA	known
Dr170	AM850012	232	Yes	Danio rerio im:7145859, transcript variant 1	ENSDARG00000020242, XM_6	mRNA	known
Dr171	AM850011	227	Yes	procollagen, type XVIII, alpha 1, splice variant	ENSDARESTG00000024579	mRNA	known
Dr172	AM850010	204	Yes	Danio rerio EST gene, mRNA	ENSDARESTG00000006086, XM	mRNA	known
Dr173	AM850009	229	Yes	IMPORTIN RAN BINDING	ENSDARG00000035751, AY28	mRNA	known
Dr174	AM850008	283	Yes	ARP3 actin-related protein 3 homolog	ENSDARESTG00000016041, NM	mRNA	known
Dr175	AM850007	250	Yes	Danio rerio si:ch211-11c20.1, mRNA, plakophilin 2a similar	ENSDARESTG00000023026, XM_6	mRNA	known
Dr176	AM850006	225	Yes	Danio rerio hypothetical protein LOC541409	ENSDARG00000038694, XM_0	mRNA	known
Dr177	AM850005	231	Yes	Danio rerio similar to cullin 3	ENSDARG00000018452, XM_6	mRNA	known
Dr178	AM850004	214	Yes	Chr:17, intron of known gene	ENSDARG00000053829, Pax9	hnRNA	known
Dr179	AM850003	255	Yes	Danio rerio cDNA clone IMAGE:7431086, partial cds	ENSDARESTG00000033158, BC	mRNA	known, Hypothetica
Dr180	AM850002	244	Yes	Danio rerio LOC563824 mRNA	ENSDARESTG00000004083, XM	mRNA	known
Dr181	AM850001	258	Yes	Danio rerio hypothetical LOC564005, some similarity to collagen	ENSDARESTG00000030156, GE	mRNA, hnRNA	known, Hypothetica
Dr182	AM850000	229	Yes	Danio rerio thyroid transcription factor 1b (titf1b), mRNA	ENSDARG00000019835, NM_1	mRNA	known
Dr183	AM849999	208	Yes	Danio rerio similar to gefiltin (LOC798092), mRNA	ENSDARESTG00000026882, XM	mRNA	known, Hypothetica
Dr184	AM849998	225	Yes	Zfin: rpsa Ribosomal protein S2	ENSDARG00000019181	mRNA	known
Dr185	AM849997	243	Yes	Chr:17 outside of any known or predicted transcription unit		NonTrans	
Dr186	AM849996	246	Yes	Zv6_NA1062:	GENSCAN00000044310	mRNA	Hypothetical
Dr187	AM849995	261	Yes	BROMODOMAIN ADJACENT TO ZINC FINGER DOMAIN 2A TRANSCRIPTION TERMINATION FACTOR I INTERACTIN	ENSDARG00000053572	mRNA	known
Dr188	AM849986	182	Yes	Danio rerio opsin 1 (cone pigments), short-wave-sensitive 1, mRNA, complete cds	ENSDARG00000045677, MGC:8	mRNA	known
Dr189	AM849994	240	Yes	Danio rerio transcription factor AP-2 alpha, mRNA, ZFIN_ID: tfap2a	ENSDARG00000059279, BC066	mRNA	known
Dr190	AM849993	225	Yes	Danio rerio alpha II-spectrin mRNA, complete cds	ENSDARESTG00000006348, EF	mRNA	known
Dr191	AM849992	245	Yes	Chr:17, outside of any known or predicted transcription unit		NonTrans	
Dr192	AM849991	204	Yes	Chr:4, inside intron of EST gene	ENSDARESTG00000027561	hnRNA	known
Dr193	AM849990	200	Yes	Danio rerio similar to magnesium-dependent phosphatase-1 mRNA	ENSDARESTG00000017443, XM	mRNA	known
Dr194	AM849989	203	Yes	Chr:15, inside intron of known gene	ENSDARG00000038377	hnRNA	known
Dr195	AM849988	242	Yes	PROLINE OXIDASE MITOCHONDRIAL PRECURSOR	ENSDARG00000044804, XM_6	mRNA	known
Dr196	AM849987	235	Yes	PREDICTED: Danio rerio similar to Cyclin G associated kinase (LOC792437), partial mRNA	ENSDARESTG00000027001, XM	mRNA	known
Dr197	AM850054	218	Yes	Chr:8, inside intron of known gene	ENSDARG00000056175	hnRNA	known
Dr198	AM850053	276	Yes	Chr:9 400 bp 3' of known gene, ZFIN_ID: ndufa10, and in intron of ab intitio gene	ENSDARG00000013333	hnRNA	known
Dr199	AM850052	256	Yes	Danio rerio zgc:110594, mRNA, similar to reticulocalbin 2	ENSDARG00000039378, zgc:11	mRNA	known
Dr200	AM850051	193	Yes	PREDICTED: Danio rerio hypothetical LOC559656, mRNA, acyl-CoA synthetase long-chain family	ENSDARG00000014674, XM_6	mRNA	known
Dr201	AM850050	233	Yes	PREDICTED: Danio rerio similar to KIAA0835 protein, myelin transcription factor 1 (MYT1)	GENSCAN00000000112, ENSDA	mRNA	known
Dr202	AM850049	293	Yes	Danio rerio zgc:158419, mRNA RAB GTPASE BINDING EFFECTOR 2 RABAPTIN 5BETA	ENSDARG00000061213, NM_0	mRNA	known
Dr203	AM850048	233	Yes	PREDICTED: Danio rerio similar to KIAA0835 protein, myelin transcription factor 1 (MYT1)	GENSCAN00000000112, ENSDA	mRNA	known
Dr204	AM850047	259	Yes	Danio rerio cDNA clone MGC:158849 IMAGE:7906912, complete cds, arginyltransferase 1 (ATE1)	BC134811.1	mRNA	known
Dr205	AM850046	196	Yes	Chr:6, in an intron	ENSDARESTG00000023065	hnRNA	known
Dr206	AM850045	201	Yes	Danio rerio glycerol-3-phosphate dehydrogenase 1a (gpd1a), mRNA	ENSDARG00000043701, NM_0	mRNA	known
Dr207	AM850044	191	Yes	Chr:21. Lies 150 bp 3' of known gene ZFIN_ID: zgc:123320, ENSDARESTG00000012970		hnRNA	known
Dr208	AM850043	193	No	Chr:7, hypothetical miRNA containing ncRNA transcription unit	ENSDARG00000063799	ncRNA, hnRNA	Hypothetical
Dr209	AM850042	211	No	Chr:5	ENSDARESTG00000012987	hnRNA	known
Dr210	AM850041	273	Yes	Danio rerio LOC555584, mRNA	ENSDARG00000062525, XM_67	mRNA	known
Dr211	AM850040	232	Yes	Danio rerio zgc:63688, mRNA GOLGIN SUBFAMILY A MEMBER 1 GOLGIN 97	ENSDARG00000008979, NM_2	mRNA	known
Dr212	AM850039	254	Yes	(LOC566553), mRNA ATP DEPENDENT HELICASE EC_3.6.1.	ENSDARG00000059556, XM_6	mRNA	known
Dr213	AM850038	217	Yes	DEAD (Asp-Glu-Ala-Asp) box polypeptide 3, ZFIN_ID: ddx3	ENSDARG00000020573	mRNA	known
Dr214	AM850037	207	No	Matches two loci equally well and behaves same in both. A2BDW8_BRARE and ptpru, mRNA	ENSDARG00000021928,ENSDA	mRNA, hnRNA	known
Dr215	AM850036	186	Yes	Chr:11, in ab initio forward frame.	FGENESH00000085417	hnRNA	Hypothetical
Dr216	AM850035	207	Yes	Chr:16, 11 - spans intron/exon boundary of Uniprot/SPTREMBL: A2BDW8_BRARE and ZFIN_ID: ptpru	ENSDARG00000021928,ENSDA	mRNA, hnRNA	known
Dr217	AM850034	283	Yes	GTPASE ACTIVATING, RhoGAP containing	ENSDARG00000052950	mRNA	known
Dr218	AM850033	274	Yes	RHO GTPASE ACTIVATING 25	ENSDARG00000061028	mRNA	known
Dr219	AM850032	233	Yes	Tropomyosin like, UVEAL AUTOANTIGEN WITH COILED COIL DOMAINS AND ANKYRIN REPEATS, ZFIN_ID: si:dk	ENSDARG00000062103	mRNA	known
Dr220	AM850031	213	Yes	ZFIN_ID: psmb7, PROTEASOME SUBUNIT BETA TYPE PRECURSOR, 20S proteasome, A and B subunits	ENSDARG00000037962	mRNA	known

Dr221	AM850030	231	Yes	200 nt downstream in forward orientation of ENSDARESTG00000014347	GENSCAN00000024218	hnRNA	known
Dr222	AM850029	215	Yes	ACYL COA BINDING ACBP, ZFIN_ID: zgc:112043	ENSDARG00000034883	mRNA	known
Dr223	AM850028	183	Yes	Dynamin, ZFIN_ID: zgc:114072	ENSDARG00000020270	mRNA	known
Dr224	AM850027	240	Yes	ARFAPTIN ADP RIBOSYLATION FACTOR INTERACTING, ZFIN_ID: arfip1	ENSDARG00000054236	mRNA	known
Dr225	AM850026	203	Yes	Dynein light chain, type 1, ZFIN_ID: dynll2	ENSDARG00000058454	mRNA	known
Dr226	AM850025	219	Yes	Matches over a hundred scattered sites with p<1E-78		NonTrans	
Dr227	AM850024	214	Yes	Matches over a hundred scattered sites with p<1E-70.		hnRNA, NonTrans	
Dr228	AM850023	276	Yes	Chr:15 outside of any known or predicted transcription unit		NonTrans	
Dr229	AM850022	188	Yes	CCR4 NOT TRANSCRIPTION COMPLEX SUBUNIT 6, ZFIN_ID: cnot6,	ENSDARG00000008255	mRNA	known
Dr230	AM850021	264	Yes	CLEAVAGE AND POLYADENYLATION SPECIFICITY FACTOR SUBUNIT 1, ZFIN_ID: cpsf1	ENSDARG00000034178	mRNA	known
Dr231	AM850020	238	Yes	Chr:5, forward orientation in known gene	ENSDARG00000035122	hnRNA	known
Dr232	AM850019	227	Yes	TRANSFERRIN RECEPTOR, ZFIN_ID: tfr1b	ENSDARG00000012552	mRNA	known
